# Effect of Dioxane and N-Methyl-2-pyrrolidone as a Solvent on Biocompatibility and Degradation Performance of PLGA/nHA Scaffolds

**DOI:** 10.52547/ibj.25.6.408

**Published:** 2021-10-19

**Authors:** Neda Aboudzadeh, Alireza Khavandi, Jafar Javadpour, Mohammad Ali Shokrgozar, Mohammad Imani

**Affiliations:** 1 Department of Materials Science and Engineering, Iran University of Science and Technology (IUST), Tehran, Iran;; 2 National Cell Bank of Iran, Pasteur Institute of Iran, Tehran, Iran;; 3 Novel Drug Delivery Systems Dept., Iran Polymer and Petrochemical Institute, P.O.Box 14965/115, Tehran, Iran

**Keywords:** Freeze drying, Porosity, Solvents

## Abstract

**Background:**

Solvent casting/particulate leaching is one of the most conventional methods for fabricating polymer/ceramic composite scaffolds. In this method, the solvent generally affects resulting scaffold properties, including porosity and degradation rate.

**Methods:**

Herein, composite scaffolds of PLGA/nHA with different percentages of nHA (25, 35, and 45 wt. %) were prepared by the solvent casting/particle leaching combined with freeze drying. The effects of two different solvents, DIO and NMP, on morphology, porosity, bioactivity, degradation rate, and biocompatibility of the resulting scaffolds were investigated.

**Results:**

The results revealed that increasing the nHA percentages had no significant effect on the porosity and interconectivity of scaffolds (*p* > 0.05), whereas altering the solvent from DIO into NMP decreased the porosity from about 87% into 71%, respectively. Moreover, scaffolds of DIO illustrated the high results of cell proliferation compared to those of NMP; the cell viability of GD25 decreased from 85% to 65% for GN25. The findings also indicated that scaffolds prepared by NMP had a higher rate of losing weight in comparison to DIO. Adding nHA to PLGA had a significant effect on the bioactivity of scaffolds (*p* < 0.05), composite scaffolds with 45 wt % nHA had at least 30% more weight gain compared to the neat polymer scaffolds.

**Conclusion:**

The DIO scaffolds have higher rates of porosity, interconnectivity, bioactivity, and biocompatibility than NMP scaffolds due to its high evaporation rate.

## INTRODUCTION

During recent years, biodegradable synthetic polymers have widely been used for the production of porous scaffolds in order to provide tissue-based artificial organs^[^^1^^-^^3^^]^. Among these polymers, PLGA, PLA, and PGA have received a great attention for biomedical applications due to their appropriate biocompatibility^[^^4^^,^^5^^]^, suitable mechanical properties^[^^4^^,^^6^^,^^7^^]^, high thermal stability^[^^8^^-^^10^^]^, and acceptable cell adhesion features^[^^7^^,^^11^^]^. These polyesters are also among the few FDA approved biodegradable polymers^[^^12^^]^. 

Different techniques have been proposed for the preparation of polymer scaffolds such as gas foaming^[^^13^^,^^14^^]^, solvent casting/particulate leaching^[^^15^^,^^16^^]^, phase separation^[^^15^^,^^17^^]^, emulsion freeze drying^[^^18^^]^ or some combinations. Among these methods, the solvent casting/particulate leaching has extensively been employed in the fabrication of temporary supports because it provides an easy control of the pore structure^[^^15^^]^. Briefly, the steps of solvent casting for scaffolds are the dissolution of the polymer in a solvent and then adding porogens or ceramic particles if intendant and finally casting the solution into a predefined three-dimensional mold. In this method, the pore structure and pore size, and also their morphology can easily be adjusted using the porogens of different particle sizes and morphologies^[^^15^^,^^17^^]^. The other notable point in this process is the solubility of polymer in solvent, which is related to polymer crystallinity, hydrophilicity, hydrogen bonding, and molecular weight. Therefore, concentration of a polymer dissolved in various solvents depends on polymer type and its molecular weight. Normally, the high molecular weight polymers tend to solidify faster and give higher solution viscosities than the low molecular weight polymers^[^^19^^]^. According to the solubility parameters of PLGA, organic solvents such as dimethylformamide, chloroform, and DIO^[^^13^^]^ should be used. However, toxicity of these solvents and their residues may negatively affect the biocompatibility and cell proliferation properties of the resulting scaffolds^[^^14^^,^^20^^]^. To resolve this problem, a technique, namely freeze drying, was combined with this method to eliminate the remnant solvent in scaffolds as far as possible.

In this research, not only a combination of solvent casting and freeze drying was used to prepare the nanocomposite scaffolds based on PLGA and nHA but also NMP was examined as a substitution solvent for the toxic solvents, which is commonly used to dissolve PLGA. NMP is a biocompatible solvent approved by FDA for parenteral applications and broadly used for the preparation of transdermal or injectable drug delivery systems^[^^18^^]^. As an example, Eligard®, an *in situ* forming implant, containing a luteinizing hormone-releasing hormone agonist, i.e. Leuprolide acetate (3% w/w), in a carrier system composed of PLGA 75/25 (33% w/w) dissolved in NMP (64% w/w) as a solvent^[^^21^^,^^22^^]^ is mostly administered for the management of advanced prostate cancer. 

In the present study, nanocomposite-based scaffolds of PLGA with various percentages of nHA and two different solvents were fabricated. The solvents used were comprised of NMP as a biocompatible solvent and DIO as a common organic solvent. The solvent casting/particulate leaching and freeze-drying technique were designated to prepare scaffolds, and finally the porous structure, bioactivity, degradation rate, and biocompatibility of the resultant scaffolds were assessed. 

## MATERIALS AND METHODS

**Materials**

Calcium chloride, sodium hydroxide, NMP, and NaCl were purchased from Merck (Darmstadt, Germany). NaCl crystals were sieved to attain a 300-500 μm size range beforehand. CMC was obtained from Sigma-Aldrich (Milwaukee, USA). Diammonium hydrogen phosphate and DIO were obtained from Scharlau (Barcelona, Spain). Chemicals were all of reagent grade and used as received without further purification. PLGA with 85:15 copolymer composition and intrinsic viscosity of 5.1 dl/g (LG 857) was procured from Boehringer Ingelheim (Ingelheim, Germany). The NHA was synthesized in house and thoroughly characterized as previously reported^[^^23^^]^. 

**Preparation of nanocomposite scaffolds**

To fabricate PLGA/nHA composite scaffolds, we combined solvent casting/particulate leaching technique with phase separation process by freeze drying. Two different solvents, NMP and DIO, were used to fabricate the scaffolds. Briefly, PLGA solutions (7% w/v) were prepared in the two mentioned solvents. NHA was dispersed in 4.5 ml of the same solvents as polymers, separately, using CMC (%1 w/v) to stabilize the dispersion. NHA dispersions were then stirred for another 30 min at 50 C and kept in an ultrasonic bath for 5 min before mixing with the polymer solutions. To include 25, 35, and 45% w/w of nHA in the final compositions, a specific volume of the ceramic dispersions was added to the similar polymer solutions. Then porogens, i.e. NaCl crystals (300-500 μm size range) were added to the mixture composed of the polymer and nHA. After that, the mixtures were thoroughly mixed in a dual centrifugal mixer (SpeedMixer^®^ DAC 150, Hauschild Engineering, Germany) at 3000 rpm for 30 seconds. The compositions were poured into a polyethylene cylinder mold and freeze-dried for 48 h. Thereafter, the nanocomposites were immersed in deionized water for 24 h to remove the embedded salt completely. The deionized water was thoroughly replaced every 6 h. Compositions of the prepared nanocomposite scaffolds are tabulated in Table 1.

**Physical characterization of the scaffolds**

To illustrate the effects of different solvents on pore morphology of scaffolds, the cross-sectional view of samples was evaluated by scanning electron microscopy (Vega II XMU, Tescan, Czech Republic). The samples firstly sputtered in liquid nitrogen, and then their surface was coated with a thin layer of gold under vacuum. Porosity of the nanocomposite scaffolds prepared via different solvents and nHA contents was determined by liquid displacement method^[^^24^^]^. Ethanol was used as the displacing liquid for the porosity structure as it effortlessly penetrates into the pores and does not induce shrinkage or swelling. First dry weight of each sample was measured, and then to gain submerged weight, the samples were submerged in ethanol for 24 h. Finally, the saturated weights of the samples were determined after they were exited from ethanol. The following formula was utilized to calculate the porosity of samples. Where M_saturated_, M_dry_, and M_submerged _stand for saturated, dry, and submerged weights, respectively. At least three specimen were examined for each presented porosity value to provide statistically consistent results.

%Porosity= (M_saturated_- M_dry_)/(M_saturated_- M_submerged_) × 100                    (1)

**Table 1:** Compositions and abbreviations of the PLGA/nHA samples

**DIO** **(ml)**	**NMP** **(ml)**	**CMC** **(g)**	**NaCl** **(g)**	**nHA** **(g)**	**nHA** **(%)**	**PLGA** **(g)**	**Abbreviation**	**Sample**
0.0	4.5	0.00	1.8	0.00	0	0.3	GN0	PLGA
0.0	4.5	0.01	1.8	0.01	25	0.3	GN25	PLGA/25 nHA
0.0	4.5	0.01	1.8	0.16	35	0.3	GN35	PLGA/35 nHA
0.0	4.5	0.01	1.8	0.25	45	0.3	GN45	PLGA/45 nHA
4.5	0	0.00	1.8	0.00	0	0.3	GD0	PLGA
4.5	0	0.01	1.8	0.01	25	0.3	GD25	PLGA/25 nHA
4.5	0	0.01	1.8	0.16	35	0.3	GD35	PLGA/35 nHA
4.5	0	0.01	1.8	0.25	45	0.3	GD45	PLGA/45 nHA

***In vitro*** **characterization of the scaffolds**

**Bioactivity assay**

The bioactivity of scaffolds were determined by measuring the weight increment of samples while were incubated in SBF. To this end, completely dried disks of the samples were first weighted (Wa) and then after sterilizing by ethanol were incubated in SBF for 48 h without any vibration disturbance. At the end, the samples were removed from the SBF and following twice washing with deionized water, were dried in a vacuum oven at 80 C for 2 h. The samples were weighed again (Wb), and according to the following formula, the percentage of weight increment was calculated:

P = (W_b_-W_a_)/W_a_ × 100                      (2)

**Cell experiments**

 The human osteosarcoma cell line (G-292 colon A141B1; no. = C 116) was obtained from the National Cell Bank of Iran, Pasteur Institute of Iran (Tehran) and was then used to investigate the efficacy of nanocomposite scaffolds prepared by different solvents and compositions. DMEM (Gibco, Scotland) supplemented with 10% v/v of FCS (Seromed, Germany) and 100 IU/ml of penicillin and streptomycin (Sigma, USA) was used for cell cultures. The nanocomposite samples (e.g. GN0, GN25, GN35, GN45, GD0, GD25, GD35, and GD45) with the size of 2 × 2 × 2 mm were prepared and sterilized by soaking into two changes of ethanol (70% v/v) for 30 min and then by soaking into three changes of PBS solution for 15 min. The samples were finally incubated in a humidified atmosphere with 5% CO_2 _content at 37C overnight. Cell proliferation assay was carried out by MTT assay. Briefly, the cells were transferred into a 96-well microtiter plate at 2 × 10^3^ cells/well and exposed to composite samples. Three wells (tissue culture polystyrene with media only) served as a negative control, and three wells, containing neat PLGA-based scaffold samples G0 (i.e. samples without any nHA), served as positive control. The plates were incubated at the same conditions as before with half media changed every day for seven days. The samples were then removed from the wells, and 10 μl of a 5 mg/ml solution of MTT (Sigma-Aldrich,) was added to each well and then incubated at 37 C for 5 h. Formed formazan crystals were dissolved by the addition of 100 μl/well of acidified isopropanol containing 0.05 N HCl (Sigma-Aldrich). Subsequently, the plates were incubated at 37 C for 10 min and changed to 4 C for 15 min before absorbance measurements. A multiwall microplate reader (ICN, Switzerland) at 570 nm was used to record the OD.

***In vitro*** **degradation test**

*In vitro* degradation test was set up to monitor the weight loss of the scaffolds in PBS medium for eight weeks. In this regard, completely dried disks were prepared from the scaffolds as previously discussed using both solvents and various contents of nHA, i.e., 25%, 35%, 45%, then were cut into eight pieces and sterilized by ethanol. Dry samples were weighed (W_a_) and incubated in PBS at 37 C in static conditions. At weekly intervals, the degradation media was renewed, and three samples of each group removed, washed 

**Fig 1:**
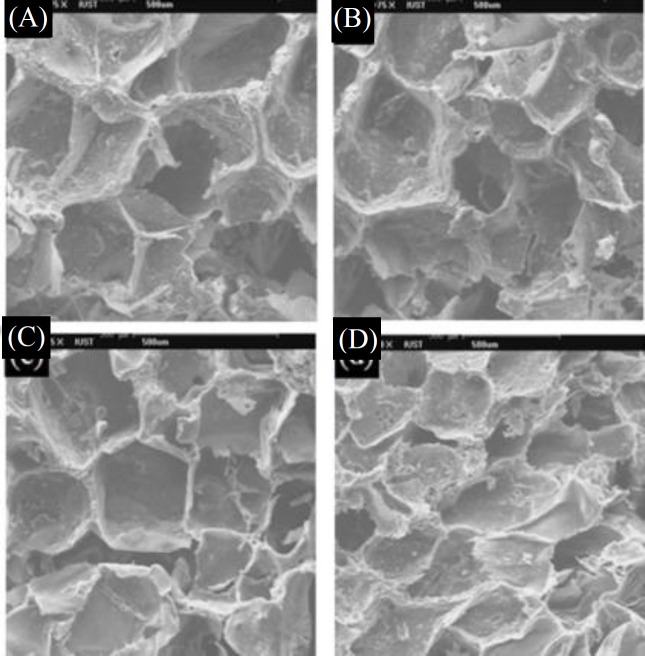
SEM micrographs of scaffolds (A) GD0, (B) GD25, (C) GD35, and (D) GD45

twice with deionized water and ethanol and dried in a vacuum oven (10^-2 ^bar, 40 C, 90 min). Samples were weighed again (W_b_), and weight loss percentage was calculated according to the formula (3): 

 P = (W_a_-W_b_)/W_a _× 100%                      (3)

**Fig. 2:**
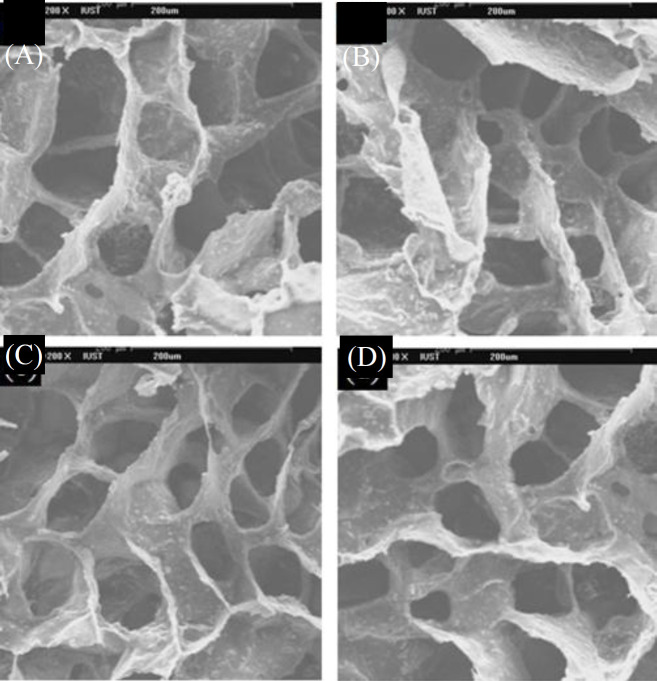
SEM micrographs of scaffolds (A) GN0, (B) GN25, (C) GN35, and (D) GN45

**Statistical analysis**

Differences of normally distributed data were analyzed by two-way ANOVA (the analysis of variance) in Minitab 19 software. The type of solvent and nHA percentages were two different independent variables, and differences considered statistically significant in *p* < 0.05, very significant in *p* < 0.01, and highly significant in *p* < 0.001.

**Fig. 3:**
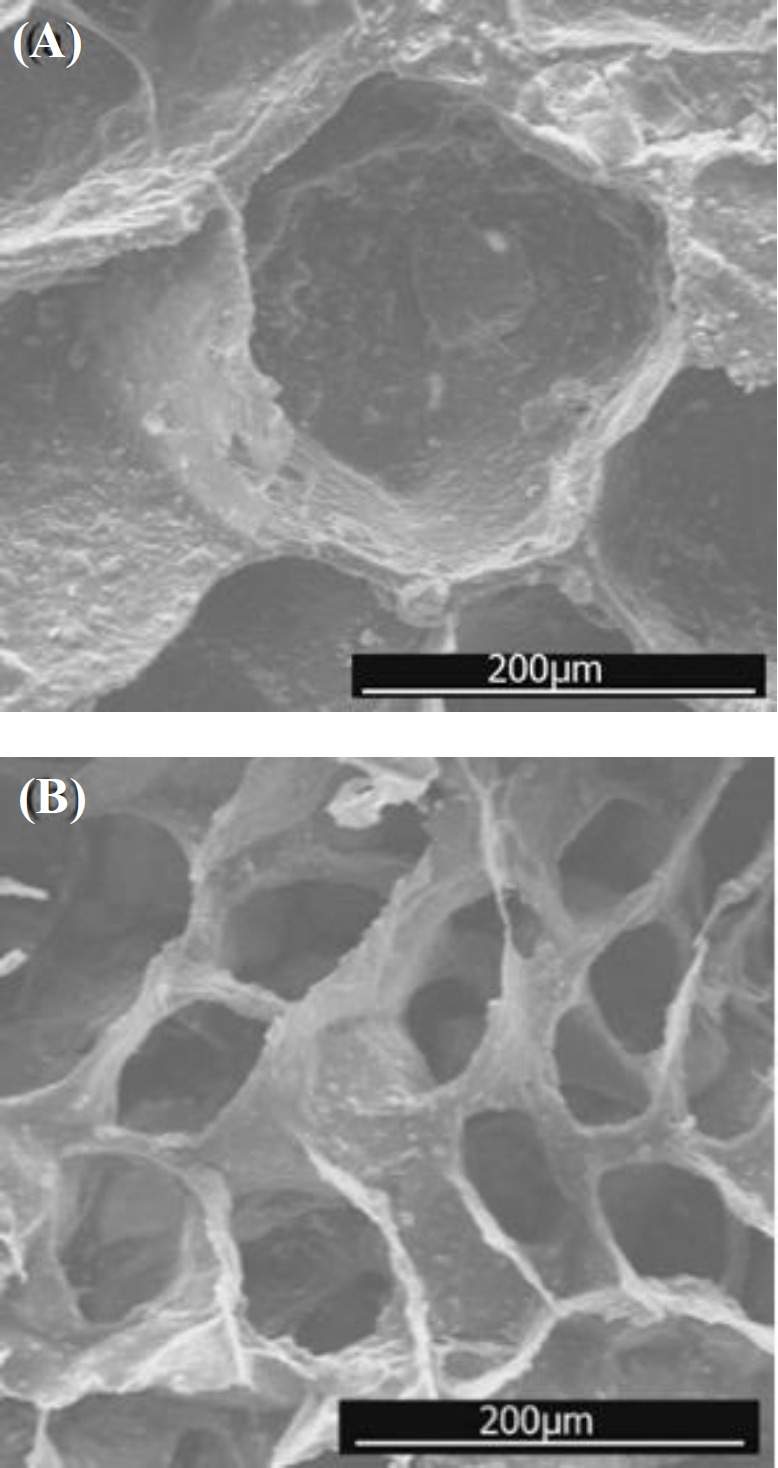
SEM micrographs of porous PLGA/nHA scaffolds by (A) NMP and (B) DIO as a solvent

## RESULTS AND DISCUSSION

**Physical characterization**

The physical characteristics of scaffolds can be described by shape, interconnectivity, average size, and size distribution of the pores^[^^24^^]^. Pore size and interconnectivity of the prepared scaffolds were studied by SEM. the increased amount of nHA into the PLGA scaffold had no significant effect on pore size and porosity (Figs. 1 and 2). SEM photomicrographs of GN25 and GD25 depicts the effect of solvent type (Fig. 3). It was observed that both groups had the pore size in the range of about 50-200 µm, which is advised for bone scaffolds^[^^25^^]^. However, the pore size for GD25 was generally smaller than GN25. A minimum pore size of >100 μm is generally required for a scaffold to be ideal in tissue engineering applications^[^^14^^,^^26^^,^^27^^]^. Proper interconnectivity between pores is also required for scaffolds to transport nutrients and waste products^[^^28^^]^; pore shape and roughness leading high cell spreading, as well^[^^29^^]^. It has been reported that any change in the fabrication process, e.g. porogen type and its percentages^[^^30^^]^, and also solvent type and its density^[^^13^^]^ can significantly affect the morphological characteristics of the scaffold. Since the solvent casting, freeze drying, and particle leaching were used for both groups of scaffolds; the difference in interconnectivity could be related to their used solvent. In solvent casting method, the solvent evaporation rate is more vital in shaping scaffold microstructure and help to have admissible porous scaffold with decent interconnection between pores^[^^31^^]^. Sander *et al.*^[^^31^^]^, prepared 75/25 PLGA scaffolds using the solvent casting/particle leaching method with three different solvents of acetone, chloroform, and methylene chloride. Scaffolds were prepared by methylene chloride showed lower interconnectivity due to its lower evaporation rate in comparison with the other solvents. Freeze drying is a processing method based on the sublimation phenomenon. In this technique, not only freeze drying properties, such as condenser temperature, chamber pressure, and freeze-drying time, can affect the properties of scaffold but also properties of used materials, such as composition, formulation, and concentration, can be significant. Therefore, the chemical composition of the used solvent can be affective. In this study, as indicated in Figure 3, the scaffold prepared by DIO as volatile solvent^[^^32^^]^ with the higher evaporation rate compared to NMP^[^^17^^]^ presented the higher porosity and interconnectivity.

**Fig. 4:**
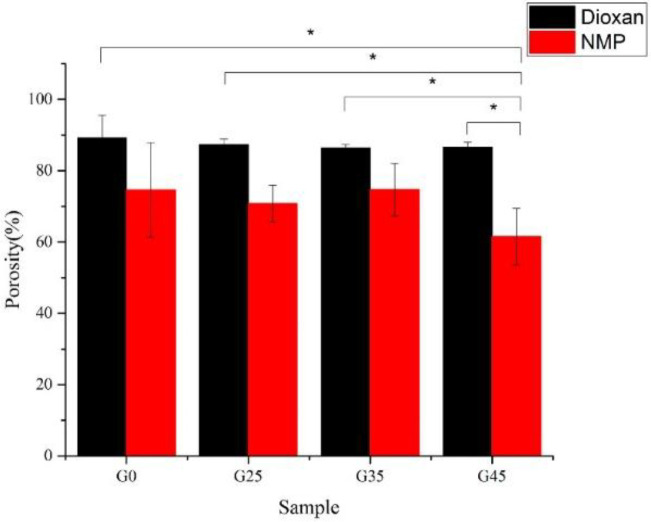
Porosity percentages of various nHAs containing scaffolds (^*^p < 0.05)

The total porosity percentage of the samples was investigated by water displacement method. The results are shown in Figure 4. Increasing the nHA content reduced the porosity of both groups of scaffolds in small quantities, although NMP scaffolds showed the more declination. The nHA particles may occupy a free space in pore and cause a reduction in the scaffold porosity^[^^13^^]^. Statistical analysis based on two-way ANOVA exposed that increasing the nHA percentages had no significant effect on the porosity of scaffolds (*p* > 0.05). Qian *et al.*^[^^25^^]^ have also noticed that adding nHA into PLGA had negligible effect on porosity. The statistical analysis also revealed that the solvent type had the high significant effect on porosity (*p* < 0.001). Comparing the porosity values of scaffolds demonstrates that scaffolds prepared by DIO have more porosity than NMP ones. Some characteristics of polymer solution like concentration and viscosity can affect the porosity and pore size of scaffold^[^^33^^]^. Consequently, the composite scaffolds prepared by NMP, compared to the DIO samples, have lower porosity. 

**Bioactivity assay**

**Fig. 5:**
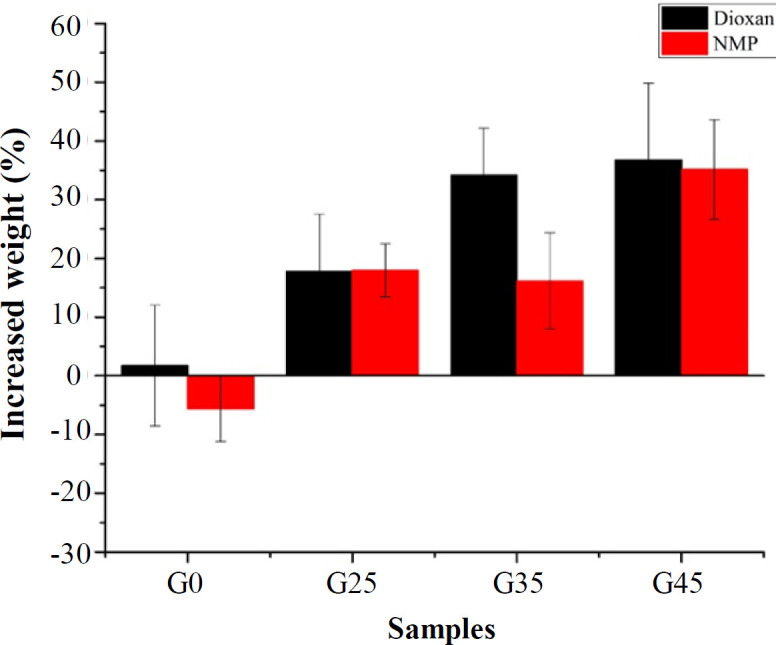
Weight gain value of the samples after incubation in SBF for 48 h. Three samples of each group were selected and examined

Formation of a biologically active bone-like apatite layer on artificial material is a necessary requirement for bonding to the living bone^[^^34^^,^^35^^]^. Bioactive materials are intended to induce a biological activity, which can lead to strong bonding to bone^[^^36^^]^. Bioactivity of GN0, GN25, GN35, GN45, GD0, GD25, GD35, and GD45 scaffolds were investigated by their incubation in SBF solution (Table 2)^[^^37^^]^. Figure 5 shows the weight changes for scaffolds after incubation in SBF for 48 h. Statistical analysis based on two-way ANOVA showed that adding nHA to PLGA had a significant effect on the bioactivity of scaffolds (*p* < 0.05). Increasing the amount of nHA as a bioactive filler increased the weight gain of the nanocomposites for both scaffold groups, which is in accordance with the results obtained by Salmasi *et al.*^[^^38^^]^. In contrast, neat samples, i.e. scaffolds without any ceramic phase (nHA), demonstrated weight loss or little weight gain in SBF in the result of polymer degradation without formation of Ca-P layer. The results also displayed that the scaffolds prepared by DIO were more bioactive than NMP, which can be attributed to their interconnected morphology; in this morphology, the more surfaces contact to SBF environment, the more weight gain^[^^39^^]^.

**Table 2:** Ionic concentration of SBF and human blood plasma

	**Ion concentration (mM)**							
	**Na** ^+^	**K** ^+^	**Ca** ^2+^	**Mg** ^2+^	**Cl** ^-^	**HPO**_4_^-^	**HCO**_3_^-^	**SO**_4_^2-^
Blood	142.0	5.0	2.5	1.5	103.0	1.0	27.0	0.5
SBF	142.0	5.0	2.5	1.5	147.8	1.0	4.2	0.5

**Degradation** **assay**

Degradation of biodegradable polyesters like PLGA mostly occurs by the uptake of water and followed by the hydrolysis of ester bonds in the initiation period. Many different factors, such as chemical composition, processing history, environmental conditions, device size and morphology (e.g. porosity), and distribution of additives or chemically reactive compounds within the matrix^[^^8^^,^^37^^,^^40^^]^, can strongly affect degradation kinetics.

**Fig. 6:**
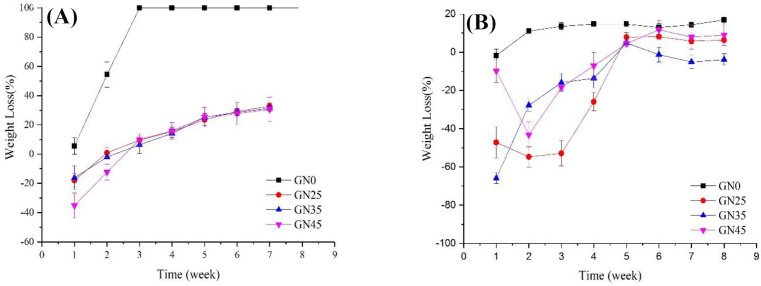
Effect of nHA content in a weight loss of nanocomposite scaffolds (a) NMP and (b) DIO as solvents

**Fig. 7:**
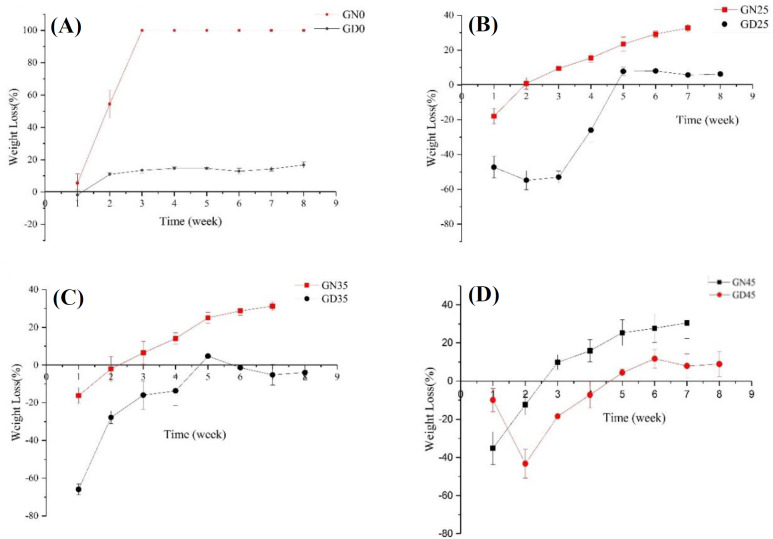
Effect of solvent on the degradation of nanocomposite for PLGA/nHA (A) 0%, (B) 25%, (C) 35%, and (D) 45%.

Herein, we separately investigated both the effects of different percentages of nHA content and solvent type on the *in vitro* degradation of each group of scaffolds; the results of losing weight of samples are depicted in Figures 6 and 7, respectively. As can be seen in Figure 6, nanocomposites of PLGA/nHA have lower losing weight rate in comparison with the neat polymer of PLGA. The acidic degradation products of PLGA accelerated the losing weight rate of scaffolds, while the presence of nHA particles in PLGA neutralized environment pH^[^^28^^,^^41^^]^. The degradation rate of nHA is inherently lower than copolymer of PLGA, and the addition of nHA to PLGA scaffold increases the corrosion resistance of nanocomposites. Moreover, nHA as reinforcement elevates the bioactivity of scaffold and motivates the formation of Ca-P layer on surface^[^^42^^]^, which restricts and declines the scaffold degradation rate. The results also show that the rate of destruction in the first two-three weeks is more severe than in the following weeks, which may be a result of morphological alterations of the polymeric structure. In the early weeks, amorphous structures were destroyed due to the easier penetration of water into them, and then the crystal structures started to break down.

Results from Figure 7 revealed that scaffolds prepared by NMP had a higher rate of losing weight in comparison to DIO, which can be attributed to the different morphologies of these two types of scaffolds. As mentioned above, the scaffolds prepared using NMP were of a closed-cell type; therefore, the acidic byproducts resulted from degradation process were accumulated possibly inside the device, which will in turn cause an autocatalytic effect^[^^43^^]^. 

**MTT assay**

**Fig. 8:**
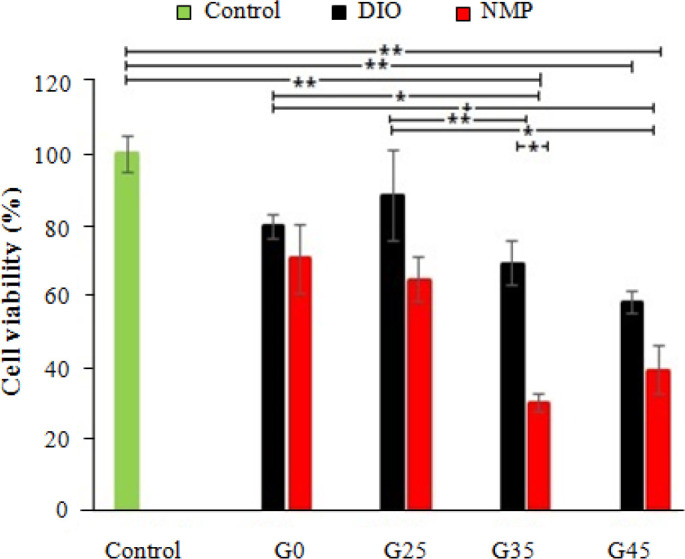
Cell proliferation assay for G-292 cells grown on nanocomposite scaffolds. (^*^p < 0.05 and ^**^p < 0.01)

Direct MTT assay was used to determine the biocompatibility of prepared scaffolds. The results compared with the control group-tissue culture polystyrene (wells containing only culture media) are shown in Figure 8. Scaffolds of DIO illustrate the high results of cell proliferation compared to those of NMP. On a macroscopic scale, the pore shape, size, and interconnectivity of scaffolds can be the significant parameters for cell seeding, mass transport, and three- dimesional tissue formation^[^^24^^,^^44^^]^. Scaffolds of DIO presented a higher explosion of osteoblasts through its porous structure. Morphologic characterization of the scaffolds of DIO was elucidated more porosity and more interconnectivity structures than those of NMP, which yield them to be more bioactivity and biocompatibility. Besides, the results of degradation tests showed scaffolds prepared by NMP had higher rates of degradation; therefore, pH of the culture medium nearby the samples may be changed at a short time and may restrict the growth of osteoblast cells^[^^45^^]^. Comparison between the biocompatibility of the scaffolds with different percentages of nHA and the same solvent demonstrates that increased percentages of nHA decreases the OD of composite scaffolds. As Aboudzadeh *et al.*^[^^23^^]^ described before, nHA particles have a high capacity for ions adsorption to form Ca-P (biologic apatite) layer; hence, a high amount of these particles disturbs the ionic equilibrium in the extracellular fluids and culture media, which significantly reduce the proliferation of osteoblast cells at *in vitro* assay. However, in the body, the circulation of fluids may lessen this ionic turbulence and its effects. Nevertheless, more cell proliferation was obtained by GN25 in comparison to GN0 (as control group) samples; therefore, more biocompatibility will be attained using nHA in polymeric structure in optimum percentages.

PLGA/nHA composite scaffolds with different percentages of nHA (0, 25, 35, and 45 wt.%) were prepared by a combined solvent casting, freeze-drying and particle leaching techniques using two different solvents consisting of NMP and DIO. The scaffolds of DIO revealed more porosity and interconnected pores in comparison with the scaffolds of NMP’s due to its high evaporation rate. The results showed that this interconnected structure in the composite scaffold has a high level of contact with SBF, which increases the bioactivity. Furthermore, adding the nHA particles to PLGA as reinforcement increase both the bioactivity and corrosion resistance of scaffolds. These nanoparticles motivate the speed of biologic apatite formation on the surface of implants. The MTT assay also revealed that nanocomposite scaffolds prepared by DIO with the optimum percentage of nHA (25 wt%) have the closet cell viability with the negative control.

In conclusion, PLGA/nHA composite scaffolds with different percentages of nHA (0, 25, 35, and 45 wt.%) were prepared by a combined solvent casting, freeze-drying and particle leaching techniques using two different solvents consisting of NMP and DIO. The scaffolds of DIO revealed more porosity and interconnected pores in comparison with the scaffolds of NMP’s due to its high evaporation rate. The results showed that this interconnected structure in the composite scaffold has a high level of contact with SBF, which increases the bioactivity. Furthermore, adding the nHA particles to PLGA as reinforcement increases both the bioactivity and corrosion resistance of scaffolds. These nanoparticles motivate the speed of biologic apatite formation on the surface of implants. Based on the MTT assay, nanocomposite scaffolds prepared by DIO with the optimum percentage of nHA (25 wt. %) have the closet cell viability with the negative control.

## CONFLICT OF INTEREST

None declared.

## References

[1] Gu Z, Kong L, Feng X, Guo T, Dai J, Li S, Huo N, Ding Y (2009). Synthesis and characterization of PLGA–gelatin complex with growth factor incorporation as potential matrix. Journal of alloys and compounds.

[2] Christy PN, Basha SK, Kumari VS, Bashir A, Maaza M, Kaviyarasu K, Arasu MV, Al-Dhabi NA, Ignacimuthu S (2020). Biopolymeric nanocomposite scaffolds for bone tissue engineering applications–A review. Journal of drug delivery science and technology.

[3] Tsang VL, Bhatia SN (2004). Three-dimensional tissue fabrication. Advanced drug delivery reviews.

[4] Cai M, Liu H, Jiang Y, Wang J, Zhang S (2019). A high-strength biodegradable thermoset polymer for internal fixation bone screws: Preparation, in vitro and in vivo evaluation. Colloids and surfaces B: siointerfaces.

[5] Ignatius A, Claes LE (1996). In vitro biocompatibility of bioresorbable polymers: poly (L, DL-lactide) and poly (L-lactide-co-glycolide). Biomaterials.

[6] Prajapati SK, Jain A, Jain A, Jain S (2019). Biodegradable polymers and constructs: A novel approach in drug delivery. European polymer journal.

[7] Sabir MI, Xu X, Li L (2009). A review on biodegradable polymeric materials for bone tissue engineering applications. Journal of materials science.

[8] Puppi D, Chiellini F, Piras AM, Chiellini E (2010). Polymeric materials for bone and cartilage repair. Progress in polymer Science.

[9] Narayanan G, Vernekar VN, Kuyinu EL, Laurencin CT (2016). Poly (lactic acid)-based biomaterials for orthopaedic regenerative engineering. Advanced drug delivery reviews.

[10] Hamad K, Kaseem M, Yang H, Deri F, Ko Y (2015). Properties and medical applications of polylactic acid: A review. Express polymer letters.

[11] Alizadeh-Osgouei M, Li Y, Wen C (2019). A comprehensive review of biodegradable synthetic polymer-ceramic composites and their manufacture for biomedical applications. Bioactive materials.

[12] Gunatillake PA, Adhikari R (2003). Biodegradable synthetic polymers for tissue engineering. European cells and materials.

[13] Choudhury M, Mohanty S, Nayak S (2015). Effect of different solvents in solvent casting of porous PLA scaffolds—In biomedical and tissue engineering applications. Journal of biomaterials and tissue engineering.

[14] Eltom A, Zhong G, Muhammad A (2019). Scaffold techniques and designs in tissue engineering functions and purposes: a review. Advances in materials science and engineering.

[15] Sola A, Bertacchini J, D'Avella D, Anselmi L, Maraldi T, Marmiroli S, Messori M (2019). Development of solvent-casting particulate leaching (SCPL) polymer scaffolds as improved three-dimensional supports to mimic the bone marrow niche. Materials science and engineering.

[16] Haider A, Haider S, Kummara MR, Kamal T, Alghyamah A-AA, Iftikhar FJ, Bano B, Khan N, Afridi MA, Han SS (2020). Advances in the scaffolds fabrication techniques using biocompatible polymers and their biomedical application: A technical and statistical review. Journal of saudi chemical society.

[17] Huang R, Zhu X, Tu H, Wan A (2014). The crystallization behavior of porous poly (lactic acid) prepared by modified solvent casting/particulate leaching technique for potential use of tissue engineering scaffold. Materials letters.

[18] Wischke C, Zhang Y, Mittal S, Schwendeman SP (2010). Development of PLGA-based injectable delivery systems for hydrophobic fenretinide. Pharmaceutical research.

[19] Al-Tahami K, Singh J (2007). Smart polymer based delivery systems for peptides and proteins. Recent patents on drug delivery and formulation.

[20] Chen J, Ye J, Liao X, Li S, Xiao W, Yang Q, Li G (2019). Organic solvent free preparation of porous scaffolds based on the phase morphology control using supercritical CO2. The journal of supercritical fluids.

[21] Sartor O (2003). Eligard: leuprolide acetate in a novel sustained-release delivery system. Urology.

[22] Astaneh R, Erfan M, Barzin J, Mobedi H, Moghimi H (2008). Effects of ethyl benzoate on performance, morphology, and erosion of PLGA implants formed in situ. Advances in polymer technology: journal of the polymer processing institute.

[23] Aboudzadeh N, Imani M, Shokrgozar MA, Khavandi A, Javadpour J, Shafieyan Y, Farokhi M (2010). Fabrication and characterization of poly (D, L‐lactide‐co‐glycolide)/ hydroxyapatite nanocomposite scaffolds for bone tissue regeneration. Journal of biomedical materials research part A.

[24] Abbasi N, Hamlet S, Love RM, Nguyen N-T (2020). Porous scaffolds for bone regeneration. Journal of science: advanced materials and devices.

[25] Qian J, Xu W, Yong X, Jin X, Zhang W (2014). Fabrication and in vitro biocompatibility of biomorphic PLGA/nHA composite scaffolds for bone tissue engineering. Materials science and engineering: C.

[26] Turnbull G, Clarke J, Picard F, Riches P, Jia L, Han F, Li B, Shu W (2018). 3D bioactive composite scaffolds for bone tissue engineering. Bioactive materials.

[27] Efraim Y, Schoen B, Zahran S, Davidov T, Vasilyev G, Baruch L, Zussman E, Machluf M (2019). 3D structure and processing methods direct the biological attributes of ECM-based cardiac scaffolds. Scientific reports.

[28] Chen L, Zhu WM, Fei ZQ, Chen JL, Xiong JY, Zhang JF, Duan L, Huang J, Liu Z, Wang D (2013). The study on biocompatibility of porous nHA/PLGA composite scaffolds for tissue engineering with rabbit chondrocytes in vitro. BioMed research international.

[29] Hutmacher DWF, Woodfield TB, Dalton PD, van Blitterswijk, Clemens A, de Boer J Scaffold Design and Fabrication.

[30] Dorati R, Colonna C, Genta I, Modena T, Conti B (2010). Effect of porogen on the physico-chemical properties and degradation performance of PLGA scaffolds. Polymer degradation and stability.

[31] Sander EA, Alb AM, Nauman EA, Reed WF, Dee KC (2004). Solvent effects on the microstructure and properties of 75/25 poly (d, l‐lactide‐co‐glycolide) tissue scaffolds. Journal of biomedical materials research part A.

[32] Wu Q, Xie W, Wu H, Wang L, Liang S, Chang H, Liu B (2019). Effect of volatile solvent and evaporation time on formation and performance of PVC/PVC-g-PEGMA blended membranes. RSC advances.

[33] Raeisdasteh Hokmabad V, Davaran S, Ramazani A, Salehi R (2017). Design and fabrication of porous biodegradable scaffolds: a strategy for tissue engineering. Journal of biomaterials science, polymer edition.

[34] Zhao C, Fan H, Zhang X (2011). Advances in biomimetic apatite coating on metal implants. Advances in biomimetics.

[35] Bosco R, Van Den Beucken J, Leeuwenburgh S, Jansen J (2012). Surface engineering for bone implants: a trend from passive to active surfaces. Coatings.

[36] Kobayashi M (2020). Enhancement of osseointegration of the hydroxyapatite implant by low intensive ultrasound wave (LIPUS) irradiation. Journal of osseointegration.

[37] Mozafari M, Salahinejad E, Shabafrooz V, Yazdimamaghani M, Vashaee D, Tayebi L (2013). Multilayer bioactive glass/zirconium titanate thin films in bone tissue engineering and regenerative dentistry. International journal of nanomedicine.

[38] Salmasi S, Nayyer L, Seifalian AM, Blunn GW (2016). Suppl-3, M8: nanohydroxyapatite effect on the degradation, osteoconduction and mechanical properties of polymeric bone tissue engineered scaffolds. The open orthopaedics journal.

[39] Dave K, Gomes VG (2019). Interactions at scaffold interfaces: Effect of surface chemistry, structural attributes and bioaffinity. Materials science and engineering: C.

[40] Heljak MK, Swieszkowski W, Kurzydlowski KJ (2014). Modeling of the degradation kinetics of biodegradable scaffolds: The effects of the environmental conditions. Journal of applied polymer science.

[41] Díaz E, Puerto I, Ribeiro S, Lanceros-Mendez S, Barandiarán JM (2017). The influence of copolymer composition on PLGA/nHA scaffolds’ cytotoxicity and in vitro degradation. Nanomaterials.

[42] Dehghanian C, Aboudzadeh N, Shokrgozar MA (2018). Characterization of silicon-substituted nano hydroxyapatite coating on magnesium alloy for biomaterial application. Materials chemistry and physics.

[43] Odelius K, Höglund A, Kumar S, Hakkarainen M, Ghosh AK, Bhatnagar N, Albertsson A-C (2011). Porosity and pore size regulate the degradation product profile of polylactide. Biomacromolecules.

[44] Chen X, Fan H, Deng X, Wu L, Yi T, Gu L, Zhou C, Fan Y, Zhang X (2018). Scaffold structural micro-environmental cues to guide tissue regeneration in bone tissue applications. Nanomaterials.

[45] Galow AM, Rebl A, Koczan D, Bonk SM, Baumann W, Gimsa J (2017). Increased osteoblast viability at alkaline pH in vitro provides a new perspective on bone regeneration. Biochemistry and biophysics reports.

